# Genetic Diversity of Cultivated Lentil (*Lens culinaris* Medik.) and Its Relation to the World's Agro-ecological Zones

**DOI:** 10.3389/fpls.2016.01093

**Published:** 2016-07-26

**Authors:** Hamid Khazaei, Carolyn T. Caron, Michael Fedoruk, Marwan Diapari, Albert Vandenberg, Clarice J. Coyne, Rebecca McGee, Kirstin E. Bett

**Affiliations:** ^1^Department of Plant Sciences, University of SaskatchewanSaskatoon, SK, Canada; ^2^London Research and Development Centre, Agriculture and Agri-Food CanadaLondon, ON, Canada; ^3^USDA-ARSPullman, WA, USA

**Keywords:** lentil, genetic diversity, population structure, germplasm, SNP markers, agro-ecological zones

## Abstract

Assessment of genetic diversity and population structure of germplasm collections plays a critical role in supporting conservation and crop genetic enhancement strategies. We used a cultivated lentil (*Lens culinaris* Medik.) collection consisting of 352 accessions originating from 54 diverse countries to estimate genetic diversity and genetic structure using 1194 polymorphic single nucleotide polymorphism (SNP) markers which span the lentil genome. Using principal coordinate analysis, population structure analysis and UPGMA cluster analysis, the accessions were categorized into three major groups that prominently reflected geographical origin (world's agro-ecological zones). The three clusters complemented the origins, pedigrees, and breeding histories of the germplasm. The three groups were (a) South Asia (sub-tropical savannah), (b) Mediterranean, and (c) northern temperate. Based on the results from this study, it is also clear that breeding programs still have considerable genetic diversity to mine within the cultivated lentil, as surveyed South Asian and Canadian germplasm revealed narrow genetic diversity.

## Introduction

Cultivated lentil (*Lens culinaris* Medikus ssp. *culinaris*) is the third most important cool-season grain legume in the world after chickpea (*Cicer arietinum* L.) and pea (*Pisum sativum* L.) (FAO, 2015). Legumes are important components in farming systems, providing environmental and ecological benefits through crop rotation, especially by contributing to soil fertility and rhizosphere diversity through biological N_2_ fixation. Global annual lentil production was around 5 million metric tons (Tg) from nearly 4.3 million ha (Mha) in 2013. Canada was the largest producer, contributing 38% of the world's production, followed by India, Turkey, and Australia (FAO, 2015). Lentil was one of the first domesticated grain legumes, originating from the Near East center of origin (Zohary, 1999). Lentil subsequently spread to central Asia and the Mediterranean Basin (Cubero, 1981; Lev-Yadun et al., 2000). It is a relatively new crop in North America, first introduced into northwest USA in the 1930s and into the northern temperate prairies of North America in the late 1960s (Muehlbauer et al., 1995). Globally today, lentil is grown in three major distinct agro-ecological zones: Mediterranean, sub-tropical savannah, and northern temperate (Tullu et al., 2011). These zones each exhibit different day lengths and temperatures, which limits the exchange of germplasm between agro-ecological adaptation zones.

Success in crop breeding is a function of heritability, genetic diversity, and selection. Natural agro-biodiversity stored in genebanks can be used to expand the diversity in crops. These collections are a vital source for discovering useful genes/alleles, which serve as a cornerstone for any pre-breeding program. There are currently 58,405 *Lens* accessions held in various genebanks worldwide (FAO, 2010). International Center for Agricultural Research in the Dry Areas (ICARDA) hosts the largest collection (19%) followed by the National Bureau of Plant Genetic Resources, India (17%) and the Australian temperate field crops collection (9%). Currently, the most accessible and accessed lentil collection is held by the USDA-ARS (United States Department of Agriculture—Agricultural Research Service; https://npgsweb.ars-grin.gov/).

Assessments of genetic diversity and relationships among preserved germplasm have important implications both for facilitating reliable documentation of genetic resources and for identifying material with possible utility for specific breeding purposes, particularly in cultivated lentil and other species with a narrow genetic base. Lentil is an autogamous diploid species with seven chromosome pairs and a relatively large genome of ~4 Gbp in the haploid complement (Arumuganathan and Earle, 1991). Considerable genetic diversity has been reported in *Lens* genetic resources for agro-morphological and phenological characteristics (e.g., Erskine and Choudhary, 1986; Erskine et al., 1989; Lazaro et al., 2001; Zaccardelli et al., 2012; Cristóbal et al., 2014). Molecular markers, as the more reliable and powerful of genetic tools, have been deployed to characterize lentil genetic resources. Various molecular marker techniques and types have been used for this purpose. These include restriction fragment length polymorphisms (RFLPs, Havey and Muehlbauer, 1989), amplified fragment length polymorphisms (AFLPs, Sharma et al., 1996; Alghamdi et al., 2014), random amplified polymorphic DNAs (RAPDs, Abo-Elwafa et al., 1995; Ford et al., 1997; Ferguson et al., 1998; Sonnante and Pignone, 2001), and inter simple sequence repeats (ISSRs, Scippa et al., 2008; Toklu et al., 2009; El-Nahas et al., 2011). Simple sequence repeats (SSRs) have the most widely used DNA markers for assessing genetic diversity in lentil (see Liu et al., 2008; Babayeva et al., 2009; Kaur et al., 2011; Zaccardelli et al., 2012; Dikshit et al., 2015; Idrissi et al., 2015).

In recent years, genome-wide nucleotide-level surveys from different individuals within or across species have received increasing emphasis (Yang et al., 2015). Development of gene-based single nucleotide polymorphisms (SNP) markers is effective for detecting genetic diversity in plant species (Frascaroli et al., 2013; Semagn et al., 2014). SNPs are the most abundant type of polymorphism in all genomes, which allows high-throughput genotyping that is low cost, locus specific, and co-dominant with simple documentation. So far, only a limited number of SNP markers have been used to study the genetic diversity in lentil (Lombardi et al., 2014; Basheer-Salimia et al., 2015). The population structure of global lentil accessions has not been extensively characterized based on their agro-ecological adaptation zones. The main aims of this study were to assess the population structure and genetic variation of a group of 352 lentil germplasm accessions of Canadian breeding lines (northern temperate adaptation) and *ex situ* germplasm collections of a diverse origin using a relatively large number of SNP markers spanning the genome.

## Materials and methods

### Plant material and DNA extraction

A total of 352 lentil accessions originating from 54 countries were collected from various sources including breeding lines obtained from the Crop Development Centre (CDC) collection in Saskatoon, Canada, from ICARDA, and the USDA-ARS. The accession numbers and origins of the selected accessions are given in Supplementary Table 1. Accessions were assigned to different major agro-ecological zones: Mediterranean, sub-tropical savannah (particularly northeast India, Nepal's lowland, and western Bangladesh), and Northern temperate according to global agro-ecological zones v3.0 (IIASA/FAO, 2012). In the Mediterranean adaptation zone, sowing occurs after the autumn equinox following a hot dry summer, prior to the winter solstice. Similarly, in the sub-tropical savannah (South Asia) planting occurs after the autumn equinox to take advantage of declining day lengths and temperatures during the juvenile phase and increases during the reproductive phase. Temperate adaptation zones require planting to occur after the spring equinox following a cold winter, prior to the summer solstice. Germplasm originating from Iran and Turkey were not assigned to specific agro-ecological zones due to different agro-ecological climates within the country borders for lentil production areas and a lack of specific collection location that would facilitate this classification. Furthermore, ICARDA breeding lines and USDA lines designated W6 # (hereafter referred to as USA breeding lines) were not assigned to an agro-ecological zones, since they were not specifically attributed a particular zone.

Canadian lines, along with ICARDA germplasm, were grown in Saskatoon, Canada and leaf tissues from at least five different plants were collected for genomic DNA extraction using a modified CTAB extraction method (Doyle and Doyle, 1990). DNA samples from leaf samples of greenhouse grown plants were provided by the USDA-ARS for the USA germplasm (PI and W6 lines; see Simon and Hannan, 1995).

### SNP discovery and genotyping

The Lc1536 GoldenGate high-throughput assay (Illumina, San Diego, CA) described by Sharpe et al. (2013) was used to genotype the 352 lentil accessions. The SNP genotyping was performed on an Illumina BeadStation 500G (Illumina, San Diego, CA) at the National Research Council (NRC), in Saskatoon, Canada with the protocol supported by Illumina (Fan et al., 2003). A robust set of 1440 of the SNP markers was used for further analyses.

### Statistical analysis

The SNP marker data were analyzed using PowerMarker v. 3.25 (Liu and Muse, 2005) to calculate minor allele frequency (MAF), heterozygosity, gene diversity, and polymorphic information content (PIC).

### Population structure and genetic diversity

The program STRUCTURE v. 2.3.4 (Pritchard et al., 2000) was used to calculate the most probable number of sub-populations (*K*) in the panel. Five independent runs were conducted for each *K* ranging from 1 to 10 with both a burn-in time and Markov Chain Monte Carlo (MCMC) replication number of 500,000 using an admixture model. Selection of the best *K*-value was based on the procedure presented in Evanno et al. (2005) by submitting the results for each *K* to the STRUCTURE HARVESTER website, which returned the *L*(*K*) and Δ*K*-value (Earl and vonHoldt, 2012), as well as a basic understanding of the nature of the germplasm. The results from STRUCTURE were presented at the country level, using the “rworldmap” package (South, 2011) in R (R Core Development Team, 2015).

Genetic structure of the lentil population was analyzed by performing principal coordinate analysis (PCoA) using GenAlEx v. 6.5 (Peakall and Smouse, 2012) based on standardized covariance of genetic distance for co-dominant markers. GenAlEx v. 6.5 was also used to calculate Analysis of Molecular Variance (AMOVA) among and within assigned groups. The genetic distance between genotypes and countries were computed using Nei's standard genetic distance (Nei, 1973) with PowerMarker software. A dendrogram was constructed from Nei's distance matrix using UPGMA and the resulting tree was visualized using iTOL v. 3.0 (Letunic and Bork, 2011).

The sequences of all the markers used in this study are described in Sharpe et al. (2013). All data are also available in KnowPulse (http://knowpulse.usask.ca) and Supplementary Table 2.

## Results

### SNP markers information

Of 1400 SNP markers, 5.46% were monomorphic among the accessions, 2.14% generated ambiguous products, and 7.21% were rejected on the grounds of excess missing data points (>30% missing data per marker). This filtering resulted in 1194 high quality polymorphic SNP markers for use in the clustering analyses. Around 40% of these SNP markers were previously mapped and shown to be evenly distributed throughout the genome (Sharpe et al., 2013). The overall PIC value was 0.3092 ± 0.0789. Measurements of the average observed heterozygosity over all loci and gene diversity were 0.0375 ± 0.0755 and 0.3932 ± 0.1160, respectively (Supplementary Table 3).

### Population structure and genetic relationship

**Figure 1:**
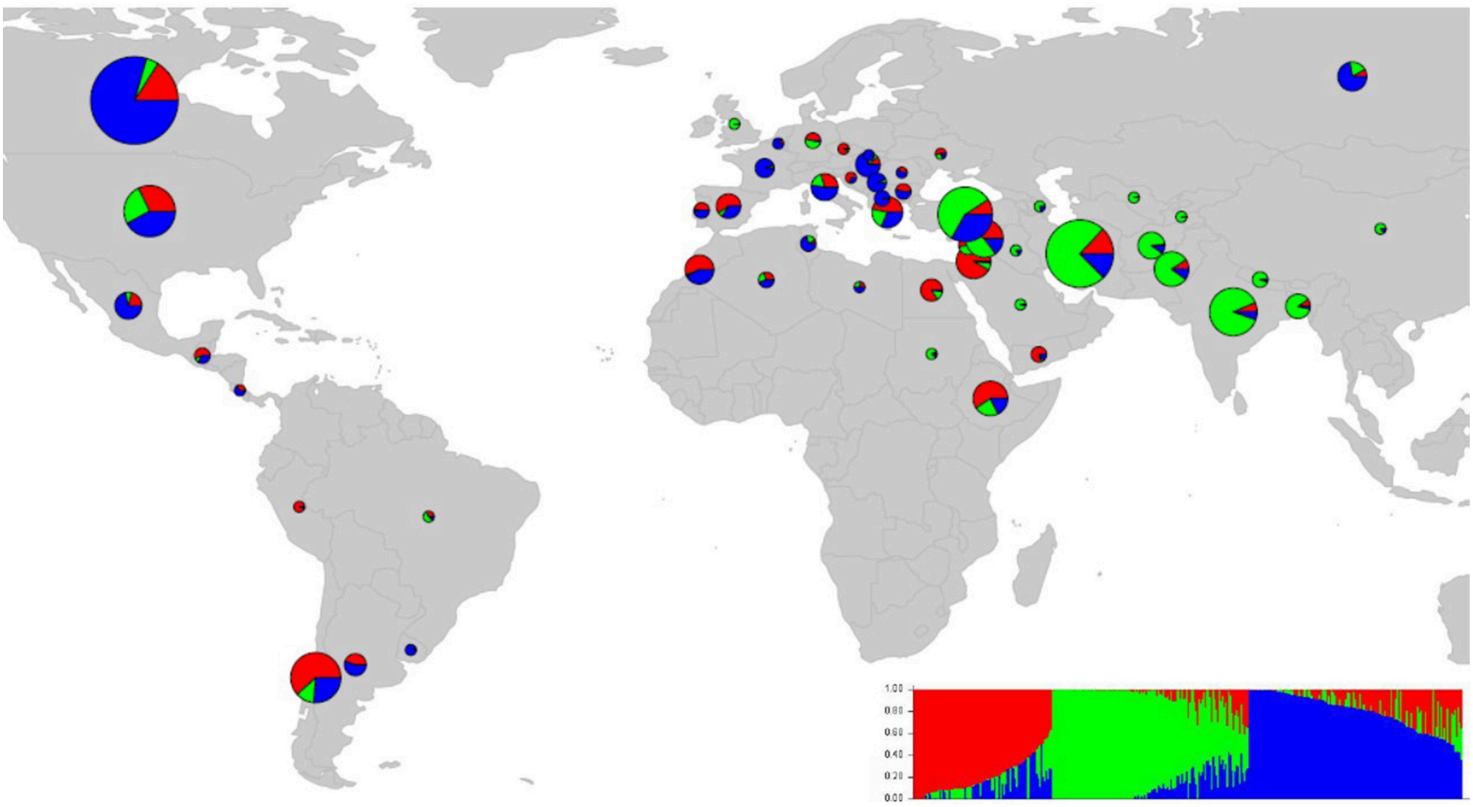
**Map of the world showing the country-specific distribution of 352 lentil accessions based on population structure (***K*** = 3)**. The size and color of pie chart is corresponding to sample size and the percentage of samples in each group, respectively.

**Figure 2:**
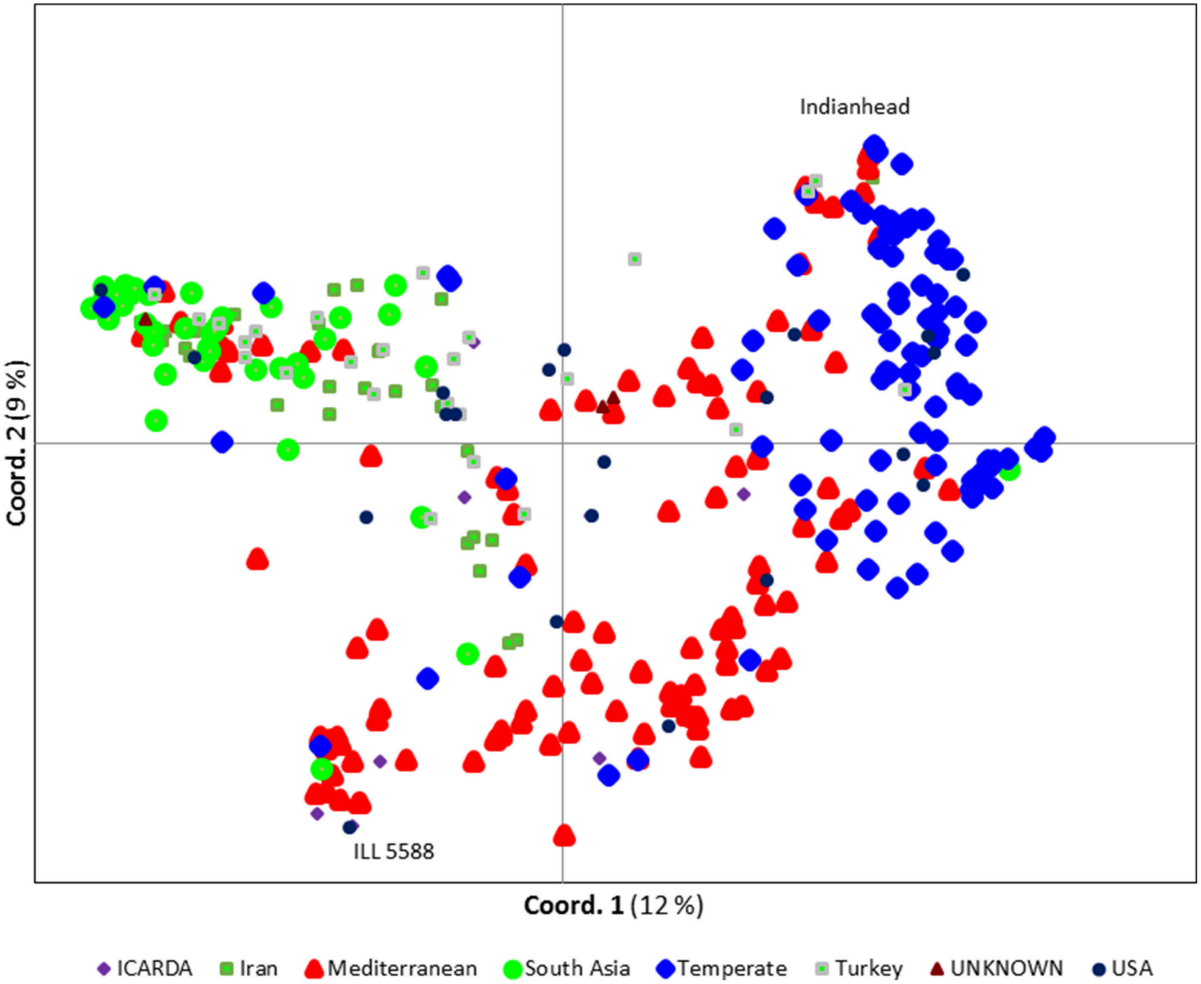
**Principal coordinate analysis (PCoA) of the 352 lentil accessions using 1194 polymorphic SNP markers**. Each symbol/color combination represents different country of origin. The most two distinct accessions, Indianhead and ILL 5588, are labeled.

We ran STRUCTURE for a range of *K* (number of fixed subgroups or cluster) from 1 to 10 on the entire set of accessions. The estimated log probability of the data [Ln*P*(*K*)] for each *K* plateaued at *K* = 3. The maximum Δ*K*-value was also reached at *K* = 3 (Supplementary Figure 1) suggesting three distinct groups. Geographical distribution of the 352 lentil accessions along with their projected population structure are shown in Figure 1. These three clusters closely reflected the origins, pedigrees and breeding history of germplasm used in this study. Accessions collected from southern Asia and the Middle East were assigned to the same gene pool. Lentil accessions originating mainly from the Mediterranean, northeast Africa (along the Nile valley from Egypt to Ethiopia), and South America were assigned to the second group, whereas the third group consisted mostly of genotypes from northern latitudes (Canada and Russian). Results from principal coordinates analysis (Figure 2) were consistent with those of STRUCTURE and UPGMA cluster analysis by revealing three clusters (Supplementary Figure 2). The AMOVA based on PhiPT-values revealed that genetic variation mainly occurred within groups (86%), while the variation between the groups was 14% (Supplementary Table 4).

Based on the 1194 polymorphic SNP markers, three pairs of accessions were genetically indistinguishable despite having different accession numbers: PI 163589 and PI 320945, PI 431675 PI 431731, and PI 297284 and PI 297285.

Based on Nei's genetic distance matrix, a close relationship exists between Indian material and germplasm from Nepal, Pakistan, and Afghanistan (0.0575, 0.0202, and 0.1310, respectively). Generally, South Asian and Middle Eastern (i.e., Iran and Turkey) germplasm grouped together, however, some of the Iranian and Turkish germplasm skewed to those from Mediterranean and northern climates. Within the second group, germplasm from Chile exhibited the closest relationship to germplasm from Morocco (0.0828). Canadian cultivars and breeding lines, representative of group 3, were closely related to much of the “W6” numbered lines which used in USDA lentil breeding program as parents in recombinant inbred lines development (USA breeding materials; 0.0968) and Russian (0.0961) germplasm (Supplementary Table 5). Most of the Canadian breeding lines were clustered together with the exception of CDC Plato, CDC Imigreen and CDC Cherie. Conversely, USA breeding lines and ICARDA breeding lines were relatively well distributed among the three groups (Figure 2).

## Discussion

The deep population structure of cultivated lentil and its importance in explaining genetic diversity underscores the value of using global lentil genetic resources to broaden the genetic base in breeding programs and to improve our knowledge of adaptation in this species. Given the sample size (individuals and country of origins) and relatively high marker density, this study identified three major clusters of germplasm reflecting the geographical origins, pedigrees, and breeding history of accessions. We categorized these clusters as (a) subtropical, South Asian, (b) Mediterranean, including Southern Europe and North Africa, and (c) northern temperate climates. These three groups reflect the main climatic regions in which lentil is widely grown as described by Tullu et al. (2011).

Lentil accessions from South Asia (primarily Nepal, India, and Pakistan) have a narrow genetic base and are genetically more isolated relative to other origins included in our study. This is likely a result of their specific phenological adaptation to the sub-tropical savannah environment and a potential genetic bottleneck during a time when lentil was introduced to South Asia around 2000 BC (Erskine et al., 1989). Most previous diversity studies of lentil diversity using molecular markers to date revealed two distinct groups: South Asian and all other origins (Ferguson et al., 1998; Alo et al., 2011; Kumar et al., 2014). In our study, however, germplasm from South Asia grouped with parts of material from Iran and Turkey, likely a stopping point on their move eastward on trade routes extending from the center of origin. A manual inspection of genetic distances between these regions shows that germplasm from Nepal, India, Afghanistan, and Pakistan are closely related, while relatively distant from Middle Eastern germplasm (Iran and Turkey). This implies there might be two distinct sub-groups within this particular cluster (South Asian and Middle Eastern). Germplasm from Afghanistan was found to be related to germplasm from South Asia as was previously reported using another set of molecular markers (e.g., Ferguson et al., 1998; Kumar et al., 2014).

Our results demonstrate that the Mediterranean, North African, and Chilean germplasm collections are similar with only a few deviations, following the classification of the Mediterranean agro-ecological zone. This is consistent with results from Ferguson et al. (1998) and Lombardi et al. (2014). Lentil was domesticated in the Eastern Mediterranean around 7–8 BC, after which it spread to Europe. Lentil was introduced to South America by the Spanish via Chile (after 1500 AD). Lentil is mainly grown as a winter crop in these regions, under conditions of declining day length followed by gradual increase in day length and temperature during the life cycle (6–7 month). This may explain the similarity among accessions of these regions.

Nearly 50% of the world's lentil production now originates from Canada (northern temperate climate) and Australia (Mediterranean climate) (FAO, 2015), regions in which lentil is a relatively new crop. Clearly, the breeding programs in these two regions have drawn from distinct global lentil genetic resources that originate in regions with climatic and growing conditions that match their local conditions. Most Canadian germplasm is related to Laird and Eston. Laird was the first Canadian lentil cultivar, released in 1978. It was a pure line selection derived from PI 343028, originally from Russia, selected for higher yield and larger seeds in the Canadian growing environment. Eston, the second cultivar released in Canada in 1980, was similarly selected for adaptation from a Turkish accession, PI 179307. The results from PCoA and genetic distance cluster analyses all demonstrate a narrower genetic variability among Canadian breeding lines. This may be attributable to relatively recent adaptation to long day northern temperate conditions in the prairies of Canada and selection pressure for improving yield and specific adaptations. A similar trend has been reported for Australian lentil breeding lines and cultivars (Lombardi et al., 2014). In contrast, breeding lines from the USA and ICARDA represent the most diverse material in this study and elsewhere (Alghamdi et al., 2014). ICARDA breeding strategies are more internationally-focused, covering a wide variety of regions and adaptive traits as part of CGIAR's policy. For example, ILL 7502 and ILL 7537 were bred for Western Asian climates, while ILL 8008 was targeted for South Asian climates (Shiv Kumar, personal communication).

Lentil domestication already has led to ~40% loss in genetic diversity (Alo et al., 2011) and breeding in specific regions has narrowed this even more. Breeding new genotypes for traits of interest requires sampling broad genetic diversity. The various statistic methods we employed here support the presence of considerable genetic variability in global germplasm that is not being accessed in some regions. The results from population structure and PCoA in this study show, to some extent, a separation by origin of the accessions with closely related pedigrees typically group together. Phenotyping of available genetic diversity has demonstrated the importance of incorporating exotic germplasm into breeding programs focusing on biotic and abiotic stresses. For example, ILL 5588 (also known as PI 592998), an ICARDA accession originally collected from Jordan, is a known source of Ascochyta blight resistance in lentil (Erskine et al., 1996). It is clearly distinct from most of the temperate germplasm (Figure 2) but it has been used in the pedigrees of some Canadian lines, including CDC Plato and CDC Cherie. This may explain why they did not cluster with the other Canadian lines.

The grouping of some accessions outside of their geographic origin may be the result of outcrossing, migration, and adaptation during cultivation of the crop by local farmers. For example, Moroccan germplasm expresses slightly less variation compared to those from Turkey due to narrower environmental conditions (Idrissi et al., 2015). The major agro-morphological changes related to adaptation are mostly improvements to yield, increasing seed size, tolerance to biotic and abiotic stresses as well as improving market-dependent quality traits. Another source of division may be the growth habit of spring and winter types, which are most adapted to different climatic regions. It has also been noted that photoperiod plays a critical role to characterize lentil cultivation areas into the respective climatic regions (Tullu et al., 2011).

The availability of EST sequences and SNP discovery are strong tools for investigating polymorphism in different species, for quantifying biological factors that influence the patterns of genetic diversity and for investigating bottlenecks due to the domestication of crop species. An allele-specific Illumina Golden Gate 1536-SNP array was constructed using SNPs discovered in expressed sequence tag (EST) sequences from nine *L. culinaris* accessions. This study has confirmed that the sub-set of SNP markers previously reported by Sharpe et al. (2013) can provide good resolution at low cost for genetic characterization of cultivated lentil germplasm in relation to the world's agro-ecological zones.

## Conclusions

Global cultivated lentil germplasm selected for this study clustered primarily based on eco-geographical origin into three basic groups: subtropical savannah, Mediterranean, and northern temperate. The narrow genetic base of some groups of germplasm (e.g., Canadian and South Asian) raises concern over the loss/penalty in yield due to biotic and abiotic stresses, particularly with the threat to global food security from climate change. This highlights the importance of harnessing the potential of lentil wild species in breeding programs by introgression of favorable genes from other regions. Based on the results from this study, it is also clear that breeding programs still have a lot of genetic diversity to mine within the cultivated species.

## Author contributions

KB and AV designed the research; HK, and CTC analyzed data; MD and MF contributed to data generation; CJC and RM contributed germplasm and reagents; HK, CTC, KB, and AV contributed to writing of the manuscript.

### Conflict of interest statement

The authors declare that the research was conducted in the absence of any commercial or financial relationships that could be construed as a potential conflict of interest.
