# Supplementary material for: Genetic Diversity of Cultivated Lentil (Lens culinaris Medik.) and Its Relation to the World's Agro-ecological Zones
Source: Front Plant Sci. 2016 Jul 26;7:1093. doi: 10.3389/fpls.2016.01093 (PMC4960256; doi:10.3389/fpls.2016.01093)
Supplement: Supplementary file 6 [file Image1.pdf]

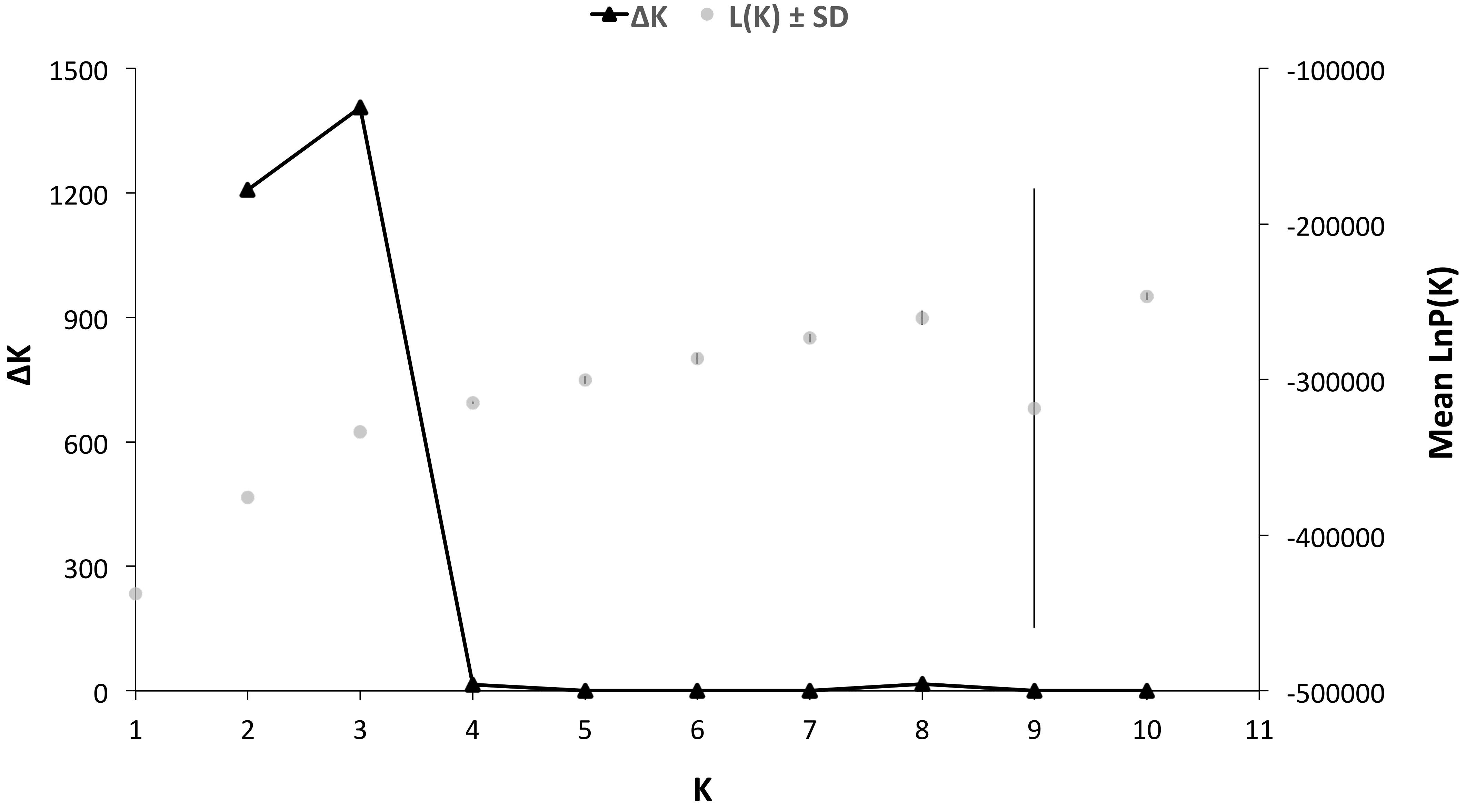

Supplementary Figure 1. Estimation of the number of population with L(K) and ΔK using STRUCTURE (K 1-10).
