# Supplementary material for: Genetic Diversity of Cultivated Lentil (Lens culinaris Medik.) and Its Relation to the World's Agro-ecological Zones
Source: Front Plant Sci. 2016 Jul 26;7:1093. doi: 10.3389/fpls.2016.01093 (PMC4960256; doi:10.3389/fpls.2016.01093)
Supplement: Supplementary file 7 [file Image2.pdf]

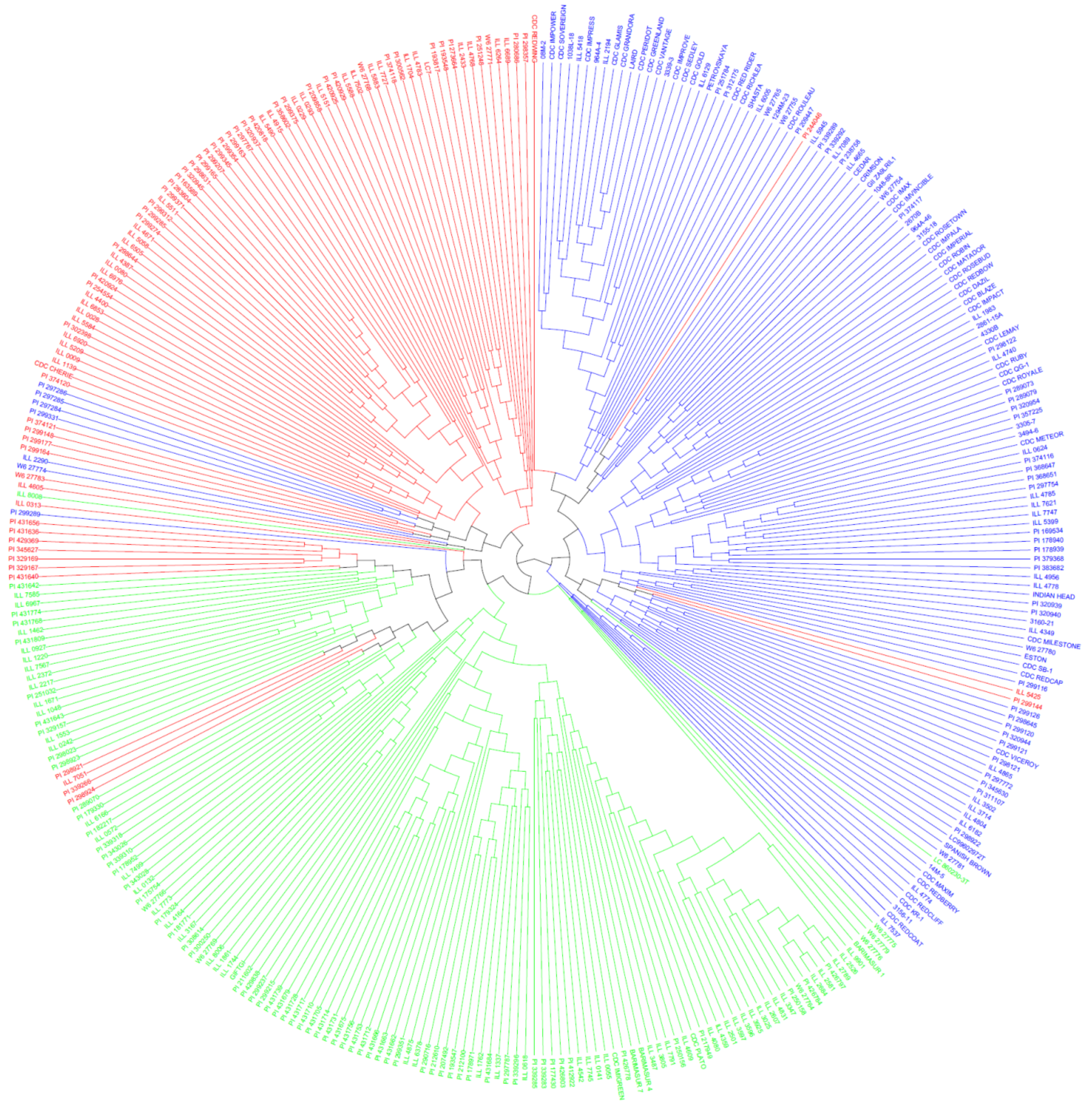

Supplementary Figure 2. UPGMA dendrogram showing relationships between individuals from 352 lentil accessions, based on 1192 SNP markers. Blue, green and red refer to accessions belong to Northern temperate, South Asia and Mediterranean groups (reflecting STRUCTURE results), respectively.
